# Sexual and reproductive health and rights and climate change about menstrual discrimination: a policy review in the context of Nepal

**DOI:** 10.3389/fgwh.2025.1560404

**Published:** 2025-06-16

**Authors:** Radha Paudel, Devaraj Acharya, Tejaswi Mili Adhikari

**Affiliations:** ^1^ Global South Coalition for Dignified Menstruation/Radha Paudel Foundation, Kathmandu, Nepal; ^2^ Research Centre for Educational Innovation and Development (CERID), Tribhuvan University, Kathmandu, Nepal; ^3^ Master of Public Health, Harvard University, Boston, MA, United States

**Keywords:** dignified menstruation, gender equality, patriarchy, power, social inclusion

## Abstract

The conversation around climate, sexual and reproductive health and rights (SRHR), and menstruation is increasing more than ever in Nepal. The discussion between climate and SRHR is more visible and it endeavors to develop the relationship between them. The claim of the role of menstrual discrimination (MD) is the construction and reinforcement of power and patriarchal demands unveiling MD as an underlying barrier for gender-responsive climate or SRHR interventions. In this vein, this study reviewed the national policies of climate change (CC) and SRHR with MD. It aims to examine the positioning of MD in ongoing policies around SRHR and CC. It is crucial to accelerate the impact of interventions related to SRHR and CC to achieve broader gender justice and human rights. The policy-related documents regarding CC and SRHR are reviewed. The visible and invisible MD is rampant across the country regardless of class, caste, region, or religion. The Government of Nepal started to work on SRHR in 2000, although the specific policies on dignified menstruation were made in 2017. MD is missed across all the conversations of CC although it talks about gender equality and social inclusion policies (GESI). The GESI policies and programs do not spell out the discrimination related to menstruation and its impact throughout life, and the SRHR policy has a similar impact. MD is one of the missing dimensions regarding power relations, patriarchy, climate, and SRHR. This study recommends a thorough unpacking of MD, including its impact, incorporating the strategies to dismantle MD in both SRHR and ensuring CC-related policies and programs for real equality and inclusion. The findings of this research become helpful to policymakers during intervention planning to overcome the situation.

## Background

Menstrual discrimination (MD) includes silence, taboos, shyness, stigma, restrictions, abuse, violence, and deprivation of services and resources that are associated with menstruation throughout the life cycle of menstruators [(1), p. 2]. Regardless of ethnicity, class, education, region, or religion, menstrual blood is considered “impure,” “dirty,” and “a state of weakness” where fear is a common driving factor to follow the practice (2). It is manifested in over 50 euphemisms and takes various forms and magnitude, including forbidding menstruators from cooking in the kitchen, from touching plants/male family members/water sources, and from participating in religious or social gatherings, deprioritizing their budget for menstrual products, and limiting their participation in daily activities, including school attendance (3–5). Such menstrual practices are a form of gender-based violence that plays a vital role in constructing and socializing the power between menstruators and non-menstruators (6). Early exposure to MD, often through witnessing discriminatory practices, reinforces a power imbalance: menstruators are socialized to view themselves as impure and disadvantaged, while non-menstruators are instilled with a sense of purity and privilege. Over time, this systemic exclusion exacerbates inequalities, leaving menstruators at a disadvantaged group and affecting all their aspects of life, which are core contributors to long-term systemic inequality, further strengthening the patriarchy. It violates at least 14 fundamental rights of the constitution of Nepal including rights to dignity, freedom, equality, health, education, food, non-discrimination, women, and safe environment (7). Since 2017, the Government of Nepal has specifically started to work to dismantle MD along with policies, namely, National Dignified Menstruation Policy (8), Menstrual Law (9), and Gender Equality Policy (10). Dignified menstruation is an innovative and holistic approach that addresses all forms of menstrual discrimination, promoting equity, dignity, and the dismantling of patriarchal norms. It seeks to transform the narrative around menstruation and its implications across all sectors of human life. The concept extends beyond conventional focus areas such as menstrual products; water, sanitation, and hygiene (WASH) facilities; taxation; period poverty; and menstrual health and hygiene management (Global South Coalition for Dignified Menstruation) (11).

Nepal disproportionately carries the burden of the impacts of climate change (CC). It is ranked the 10th most CC-affected country in the world by the Climate Risk Index, despite only contributing 0.027% to global greenhouse gas emissions (12). As natural resources are strained, humanity faces severe water scarcity, food insecurity, health risks, and poverty and displacement (13). It is imperative to recognize how both the burdens and impacts of CC unfairly fall on certain communities. This is most commonly presented through the framework of CC, which recognizes the disproportionate impact CC poses on low-income communities and the most vulnerable people particularly concerning MD that has contributed to systemic inequalities within societies. These vulnerabilities are not merely environmental but are rooted in systemic inequities, especially the issue of MD. Menstruators have often been left aside or less prioritized due to such systemic inequalities, e.g., during the COVID-19 pandemic, 70% of frontline workers were female, many of whom were compelled to wear menstrual pads for >12 h due to the lack of time to change personal protection equipment during their shifts. In some cases, they even used contraceptives to stop menstruation (14). In a context where MD is a deeply embedded socioeconomic and political barrier, the intersection of climate vulnerability and MD demands urgent attention.

The sexual and reproductive health and rights (SRHR) has been a priority of the Government of Nepal since 1998 and is a guaranteed constitutional right (7). The Government of Nepal developed the policies, including the National Adolescent Health and Strategy 2000, Legalization of Abortion 2002, and Safe Motherhood and Reproductive Act 2018. MD is prevalent in all communities in all diverse settings (15). Menstrual discrimination is a fundamental social barrier exacerbating the disproportionate impact of CC on menstruators. Menstruators, mostly women and girls, are displaced due to CC and disasters worldwide, and they are up to 14 times more likely to die in climate-related disasters than men (16).

There is a Nepali saying, “pouring water in the sand,” which is used to describe the fruitless process of acknowledging the symptom of an issue without addressing its root cause. A similar thing can be said of how MD is largely unaddressed, both in policy and activism around SRHR and CC. As the impacts of CC worsen and Nepal confronts its highly climate-vulnerable future, the interlink between climate impacts and MD as a tool propagating social inequality has become an increasingly underreported topic. Although the framework of SRHR has increasingly become a more widely acknowledged topic, MD as a barrier to accessing SRHR, and the interlink of both with CC, has not. The gender equality and social inclusion policies (GESI) policies are considered as an assumption of addressing the needs and priorities of menstruators where MD is ignored and unreported (14).

## Methods

We used secondary sources, such as policies, acts, regulations, and programs, in Nepal related to CC, SRHR, and menstruation. The inclusive criteria are MD, dignified menstruation, CC, and SRHR. These sources are based on published and unpublished articles, policies, and other documents related to the issues. For online articles, different databases were used to search the articles/reports/documents such as PubMed, ProQuest, DOAJ, Hinari, ResearchGate, Academia, and Google Scholar. Unpublished articles and data were obtained from the Radha Paudel Foundation's Library, National Planning Commission, and Tribhuvan University Central Library. The findings were based mostly on policy-related documents and articles available from 1995 up to 2024 because the Government of Nepal changed the women's and human rights course in Nepal since promised in the 1995 Beijing conference.

A total of 25 documents were identified, among which nine specific policy documents were fully reviewed. Of these, six were related to CC: Paris Climate Agreement (17, 18), Climate Change Financing Framework 2017 (19), National Climate Change Policy 2019 (20), Second Nationally Determined Contribution (NDC) 2020 (21), National Adaptation Plan 2021 (22, 23), and Vulnerability and Adaptation Assessment of Climate Sensitive Diseases and Health Risks 2022 (24). The rest of the policy documents were related to SRHR: National Adolescent Health and Strategy (2000), Legalization of Abortion (2002), and Safe Motherhood and Reproductive Act (2018) (Ministry of Health). When none of the policies met the criteria, they were reviewed based on whether they mentioned and included SRHR or acknowledged or addressed “gender” or “GESI” as a priority area, using gender or GESI as a proxy for SRHR and CC although none of the GESI policies address the menstrual discrimination.

Thematic analysis was performed, and major themes such as MD, dignified menstruation, SRHR, and CC were discussed. To investigate the policy gaps on climate justice and SRHR in relation to prevalent MD, each of the identified policy actions was reviewed under the values and principles of dignified menstruation as pioneered by the Global South Coalition for Dignified Menstruation/Radha Paudel Foundation. This included, but was not limited to, (1) explicit references to MD including the mention of any forms of MD; (2) inclusive, informed language and terminology related to MD or dignified menstruation; (3) an understanding of the complexity of MD including consider as a social barrier perpetuating menstruators' vulnerability to climate impacts; and (4) inclusion of indicators of dignified menstruation in pre and post-climate disaster relief strategy.

### Findings

#### Menstrual discrimination and dignified menstruation in Nepal

The Supreme Court identified visible forms of MD as a violation of human rights and asked the Ministry of Women to act on it in 2005 (25). It is further highlighted that the Ministry of Women, Children and Senior Citizens, Government of Nepal, formulated the guideline on the elimination of the practice of using menstrual huts during menstruation, but it was not effective due to the linear approach. This guideline was superficial, failed to acknowledge the visible and invisible forms of MD across Nepal, and misinterpreted and misled the menstrual movement. The word Chhaupadi means menstruation in the local Achhami Nepali language which is used in only a few places in west Nepal (Global South Coalition for Dignified Menstruation) (26). This report further explained that the separation of menstruators depends on the geography and economic status, e.g., in Achham, menstruators are separated into huts, and in Kathmandu, the capital city of Nepal, they are separated into the corners of the room. In both conditions, they have been following dozens of similar forms of restrictive practices such as not entering the kitchen, not touching fruits or vegetables, and not participating in cultural activities. In 2017, the Government of Nepal developed a holistic policy (directed to both visible and invisible forms of menstrual discrimination) on dignified menstruation (8). In alignment with dignified menstruation, the Menstrual Law stipulates penalties of 3 months in jail, a $30 bail, or both against any form of menstrual discrimination (9). Dignified menstruation has been incorporated into the Gender Equality Policy 2021 (10) and 16th Five-Year Periodic Plan 2024–2029 (27), and the national assembly unanimously endorsed the resolution motion on dignified menstruation on 21 March 2025 (28).

#### The SRHR discourse in Nepal

The Family Health Division, Ministry of Health, Government of Nepal, started to work around SRHR in 1998 by formulating the Safe Motherhood policy (29). Consecutively, policies, namely, National Adolescent Health and Strategy 2000 and Safe Abortion Law 2002, have taken place (30). The Safe Motherhood and Reproductive Act (2018) is promulgated and enforced as an SRHR-related program in Nepal (31). Menstruation is included as simple as a developmental milestone including the anatomy and physiology of reproductive organs. There is no space to explore the menstrual practices including the link with MD in relation to claiming the SRHR services (Table 1).

**Table 1:** SRHR policies in Nepal.

SRHR policies	MD, dignified menstruation, and CC
National Adolescent Health Strategy (30)	_
Safe Abortion Law (Ministry of Health and Population, Family Health Division, 2002)	_
Safe Motherhood And Reproductive Health Act 2018 (29)	The Word of Menstrual Care Mentioned Miscellaneous, Section 8.29

#### Climate change in Nepal

Climate Change Financing Framework 2017 (9.4.3) used the word “gender” while referring to training on CC financing to the focal person (19). The National Climate Change Policy 2019 mentioned the GESI under the objective (g) section and used the GESI as an inter-thematic area where only the word “pregnant women” was used while describing vulnerable groups. The rest of the document does not even spell out the words “menstruation,” “MD,” or “SRHR” (20). Likewise, the Second Nationally Determined Contribution 2020 (21) also did not mention MD or SRHR except for the word “GESI” as a target for participation and disaggregated data (21). One out of three goals of the National Adaptation Plan 2021 forwarded a goal where GESI included livelihood and governance (22). It allocated $0.7 billion for primarily addressing the hazards or conditions that occurred due to diseases due to CC and climate disasters. There is no mention of menstruation, MD, or SRHR directly and indirectly at all. The Vulnerability and Adaptation Assessment of Climate Sensitive Diseases and Health Risks (Ministry of Health and Population, Government of Nepal, 2022) included few reproductive health outcomes such as preterm delivery, congenital birth defects, poor nutritional status, gender-based violence under Section 5.5.1. as health inequality gaps due to CC and climate-induced disasters while explaining the vulnerable population (32) (Table 2).

**Table 2:** CC policies in Nepal.

CC policies	MD, dignified menstruation, and GESI
Paris Agreement 2015 (17, 18)	–
Climate Change Financing Framework 2017 (19)	Section 9.4.3 used the word “gender focal person”
National Climate Change Policy 2019 (20)	Used the word “GESI” twice under objective g and under inter-thematic area while explaining the vulnerability like pregnancy
Second Nationally Determined Contribution 2020	GESI used while explaining the target group
National Adaptation Plan (22)	GESI used while explaining livelihood and governance
Vulnerability and Adaptation Assessment of Climate Sensitive Diseases and Health Risks 2022	Section 5.5.1 included a few reproductive health outcomes such as preterm delivery, congenital birth defects, poor nutritional status, and gender-based violence

## Discussion

### Menstrual discrimination and its impacts

MD is manifested in over 50 euphemisms across Nepal, regardless of class, education, religion, or region. It is considered a biological tribal marker that has been historically weaponized to create social and political hierarchies and plays a key role in the construction of power relations and patriarchy. Menstruators at the age of 6–9 years have been systematically categorized as weaker, inferior, and impure compared with non-menstruators who are positioned as superior due to their absence of menstruation (6). This categorization extends beyond simple biological differences to serve as a foundational element of gender-based stereotypes across cultures, communities, and institutions including climate disasters. This false narrative and associated discriminatory practices manifest through a sophisticated network of sociopolitical controls, ranging from visible explicit violence to invisible forms of violence, all working to construct and maintain unequal power dynamics between menstruators and non-menstruators (26). MD is an underlying cause for systemic inequalities, gender stereotypes, and patriarchy that affect all life aspects of menstruators including SRHR, CC, and climate disasters. The complex and multifaceted nature of MD and its role in power relationships and patriarchy is yet to be explored and addressed. The menstruators are potential beneficiaries around the SRHR, CC, and any other sectors. In this vein, MD needs to assess across the sectors throughout the program cycle (33).

### Accelerate the vulnerability with unaddressed menstrual discrimination

The learning and socializing age of 6–9 years for MD is a departure point for power relationships and patriarchy which goes throughout the life cycle and makes a vicious circle. The underlying impact of MD directs all decisions related to agency and bodily autonomy among menstruators and also facilitates for same from non-menstruators which manifests as multiple levels of oppression. Such psychological conditioning continuously applies to the entire SRHR and CC planning including disaster planning and response. Menstruators have often been left aside or less prioritized due to such systemic inequalities, e.g., during the COVID-19 pandemic, 70% of frontline workers were female, many of whom were compelled to wear menstrual pads for >12 h due to the lack of time to change personal protection equipment during their shifts. In some cases, they even used contraceptives to stop menstruation (14). How MD serves as an embedded barrier, making menstruators more vulnerable to SRHR and CC, must be acknowledged, especially in sections of policy reports that already discussed the factors of women and girls' disproportionate vulnerability to climate change, such as Section 5.5.1 of the Vulnerability and Adaptation Assessment 2022. This must be explicitly included, not only to avoid reinforcing a traditional narrative of silence surrounding menstruation but also to ensure that the needs of menstruators are not overlooked amid an increased frequency and scale of climate impacts in the coming days. Nepal contributes the least to climate change but is the most affected, where climate change is a question of disaster preparedness and climate impact adaptation. In post-disaster contexts, issues such as food insecurity, damage to healthcare facilities, and limited access to healthcare (including menstrual products) contribute to the worsening SRHR. MD already imposes physical, mental, and emotional stress on menstruators, and in disaster contexts, this strain increases exponentially.

### Menstrual discrimination is missed in the SRHR discourse

This study reveals that the Government of Nepal has been working hard around SRHR to translate the commitment of the International Conference on Population and Development 1994 and 1995 Beijing conference and continue to achieve the targets of Sustainable Developmental Goals 2015. Since the country has struggled with visible and invisible forms of MD in various names, forms, and magnitude, it is missing across the conversation of SRHR including the policies and strategies. Wilson et al. (34) also argued that menstrual health and SRHR have been missing the opportunity to create synergy. They also revealed that the international big funders, researchers, and actors including WHO missed MD in SRHR strategies, guidance, and resources as an integral element of SRHR. The discussion on biological facts of reproductive organs does not unveil the complex MD (35). The absence of MD represents a critical gap in addressing critical issues of SRHR (36). This is further agreed by the study conducted by the Radha Paudel Foundation (15), which explained that menstrual discrimination is a barrier within a person and in family, community, schools, and health facilities for utilizing SRHR services.

### Climate change affects the menstruators

The menstruators experience systemic inequalities due to unrecognized and unaddressed MD. They face additional health challenges due to the impacts of CC including climate disasters, e.g., increased temperatures leading to alterations and irregularities in the menstrual cycle. Stress-induced hormone imbalances, which are likely to increase with the strain of natural resources and the increase of natural disasters, have also been found to disrupt the menstrual cycle and affect fertility and well-being. MD is a common and untouched underlying barrier for SRHR and climate.

The conversation on interconnection between SRHR and CC is increasing in Nepal. The SRHR health impact of climate change is taken seriously, e.g., the rising temperature impacts maternal and child health in Nepal as well. Despite having such intense visible and invisible forms of MD across the country, MD is absolutely missing in the discourse of SRHR and CC as an underlying factor for systemic inequalities and symptomatic harm. During the earthquakes of 2015 and 2023 in east and west Nepal, respectively, menstruators were not allowed to touch water sources due to menstruation, and many relief packages did not include menstrual products. In cases where menstrual products were included, they primarily consisted of disposable menstrual pads in plastic packaging. Moreover, 90% of disposable menstrual pads is another factor that contributes to the climate crisis due to the plastics and chemicals they contain (37). There is a clear gap in the connection of MD with climate change and SRHR. The departure point of power relations and patriarchy is the age of knowing something about menstruation at first in life, which plays a vital role in the unequal power relationship and patriarchal structure as well as the source of oppression and multiple forms of discrimination throughout life that needed to be acknowledged (6). In the absence of MD, policies and programs fail to address the causes of climate change and dismantle the root causes of systemic inequalities and co-occurrence of violence. While climate policies discuss a lot about GESI policies as a proxy for addressing MD, the GESI policies remain silent on MD.

### Limitations

Primarily it is based on secondary data, such as policies around SRHR, CC, and dignified menstruation in Nepal. The scope of the policy review could have been widened by including relevant policies from other countries or UN policies, but this study focuses on Nepal, so Nepalese documents are important for the analysis.

## Conclusion

MD, SRHR, and CC are deeply connected. Acknowledging and addressing MD is essential for creating equitable climate policies and SRHR. The impact of CC could be minimized if the nature of MD is acknowledged and addressed. Ensuring menstrual dignity and SRHR must become a priority in both policy and humanitarian responses, especially in vulnerable countries such as Nepal.

Menstruators' dignity and rights to freedom, equality, food, health, etc. are more vulnerable to climate-related stressors. Menstruators are often excluded from decisions about land and resources. After climate impacts, menstruators are left in vulnerable situations, yet the role of menstruation in this vulnerability remains largely unaddressed. Menstrual dignity is more than just access to products or knowing the anatomy of reproductive organs; it includes breaking all forms of MD that deny menstruators their basic rights. Climate justice cannot be fully realized without addressing these social barriers created by MD. Existing structural barriers such as MD, coupled with the increased frequency of climate disasters, make it imperative to include menstrual dignity in climate policies.

While addressing climate resilience in Nepal, it should be considered beyond infrastructural or economic solutions. It requires an explicit recognition of how social inequities, such as MD, exacerbate the impacts of climate change on vulnerable populations. The growing climate crisis underscores the need for holistic and inclusive policies that integrate menstrual dignity into SRHR, disaster preparedness, and climate adaptation strategies, ensuring that no one is left behind.
